# Pharmacokinetic boosting of olaparib: Study protocol of a multicentre, open-label, randomised, non-inferiority trial (PROACTIVE-B)

**DOI:** 10.1016/j.conctc.2025.101477

**Published:** 2025-03-29

**Authors:** Joanneke K. Overbeek, Niels A.D. Guchelaar, Ma Ida Mohmaed Ali, Muriëlle Sark, Carolien Hovenier, Wietske Kievit, Marjolijn J.L. Ligtenberg, Petronella B. Ottevanger, Haiko J. Bloemendal, Stijn L.W. Koolen, Ron H.J. Mathijssen, Ingrid A. Boere, Alwin D.R. Huitema, Gabe S. Sonke, Frans L. Opdam, Rob ter Heine, Nielka P. van Erp

**Affiliations:** a Department of Pharmacy, Radboud University Medical Center, Nijmegen, the Netherlands; b Department of Medical Oncology, Erasmus MC Cancer Institute, Erasmus University Medical Center, Rotterdam, the Netherlands; c Department of Pharmacy & Pharmacology, The Netherlands Cancer Institute, Amsterdam, the Netherlands; d Patient Advisory Group, Breast Cancer Research Group and Dutch Breast Cancer Association, Amsterdam, the Netherlands; e Stichting Olijf, Utrecht, the Netherlands; f Department for Health Evidence, Radboud University Medical Center, Nijmegen, the Netherlands; g Department of Human Genetics, Radboud University Medical Center, Nijmegen, the Netherlands; h Department of Pathology, Radboud University Medical Center, Nijmegen, the Netherlands; i Department of Medical Oncology, Radboud University Medical Center, Nijmegen, the Netherlands; j Department of Hospital Pharmacy, Erasmus University Medical Center, Rotterdam, the Netherlands; k Department of Pharmacology, Princess Máxima Center for Pediatric Oncology, Utrecht, the Netherlands; l Department of Clinical Pharmacy, University Medical Center Utrecht, Utrecht University, Utrecht, the Netherlands; m Department of Medical Oncology, The Netherlands Cancer Institute, Amsterdam, the Netherlands

**Keywords:** Clinical trial, Olaparib, Cobicistat, Pharmacokinetic boosting, Non-inferiority

## Abstract

**Background:**

Pharmacokinetic (PK) boosting is the intentional use of a drug-drug interaction to enhance systemic drug exposure. PK boosting of the anticancer drug olaparib, a CYP3A-substrate, has the potential to reduce PK variability, side effects and financial burden associated with this drug. After establishing adequate pharmacokinetic exposure with boosting in the PROACTIVE-A study, the PROACTIVE-B study is designed to evaluate non-inferiority for both efficacy and toxicity of the boosted therapy compared to the standard monotherapy of olaparib.

**Methods:**

The PROACTIVE-B study is a nationwide, multicentre, prospective, randomized, non-inferiority trial. A total of 142 patients (128 patients with BRCA+, high-grade, FIGO III/IV ovarian cancer who receive olaparib as maintenance therapy; 14 patients with other approved indications for olaparib) who start olaparib treatment in line with the drug label will be randomized between the standard monotherapy of olaparib 300 mg twice daily (BID) and the boosted therapy of olaparib 100 mg BID with cobicistat 150 mg BID. The co-primary objectives are tolerability (dose reductions due to toxicity), and efficacy (progression-free survival at 12 months) in the ovarian cancer population. Secondary objectives include health status (EQ-5D-5L), patient satisfaction (Cancer Therapy Satisfaction Questionnaire (CTSQ)), and cost effectiveness using the institute for Medical Technology Assessment (iMTA) Productivity Cost Questionnaire (iPCQ) and iMTA Medical Consumption Questionnaire (iMCQ).

**Discussion:**

PK boosting of olaparib is a potentially valuable strategy to reduce the olaparib dose and the variability in olaparib exposure with fewer side effects. Moreover, the lower costs related to the boosted therapy contribute to a durable and accessible anticancer treatment for all patients.

**Trial registration:**

The PROACTIVE study has been published at ClinicalTrials.gov under NCT05078671 on October 14, 2021 and at EudraCT under 2021-004032-28 on August 24, 2021.

## 1 Background

The accessibility to novel cancer treatments is at risk due to the increasing costs of newly approved drugs in combination with the rising incidence of cancer [1,2]. Hence, serious efforts should be made to keep anticancer treatment affordable and accessible for all patients.

Poly-adenosine diphosphate ribose polymerase (PARP)-inhibitors are a family of highly effective oncolytic drugs. PARP-enzymes play a key role in the repair of single strand DNA-breaks. Inhibition of PARP causes accumulation of single strand breaks and eventually apoptosis through synthetic lethality when double strand break repair mechanisms are inadequate [3]. Olaparib is a PARP-inhibitor, initially approved for the maintenance treatment of women with platinum-sensitive relapsed, Breast Cancer gene (BRCA)-mutated, high grade serous epithelial ovarian, fallopian tube, or peritoneal cancer, following a response to platinum-based chemotherapy [4,5]. Over the last years, olaparib has also been approved for the treatment of other stages of ovarian cancer, and subtypes of breast, pancreatic and prostate cancer, with more indications currently under investigation [6,7].

Olaparib is expensive with monthly expenses of $20,000 or €6,000 [[8], [9], [10]]. The increasing number of patients eligible for treatment with PARP-inhibitors has a substantial impact on health care expenditures [8,11]. Therefore, strategies are required to limit the impact of drug costs on health care budgets while facilitating global access to new and innovative therapies [12].

We propose pharmacokinetic (PK) boosting to reduce the dose of expensive anticancer drugs while improving the safety profile and maintaining efficacy for our patients. PK boosting is the lay term for administering a non-therapeutic active strong inhibitor of a metabolic enzyme, for example cytochrome P450 3A (CYP3A), together with a therapeutic drug that is metabolized by the same enzyme. For the treatment of Human Immunodeficiency Virus (HIV)-infection, PK boosting is routinely used in millions of people worldwide for over two decades and can, therefore, be considered as a feasible and safe approach to implement in practice [13,14]. Since many oral targeted oncolytic drugs are metabolized by CYP3A iso-enzymes, boosting could be a valuable tool to reduce the dose and, thereby, costs for many more anticancer drugs [15]. PK boosting will increase the bioavailability, due to pre-systemic CYP3A-inhibition, and decrease the elimination, due to systemic CYP3A-inhibition, of drugs that are substrates of this enzyme [13]. Therefore, the dose of a boosted drug may be reduced without compromising effective exposure. Additionally, inhibition of CYP3A could lead to less PK variability since metabolic capacity is a prominent cause for variability in systemic exposure [14]. Olaparib shows a wide variability in plasma concentrations which might be reduced by CYP3A-inhibition [16]. Predictable olaparib exposure will reduce the number of patients who are either unintentionally undertreated, or overtreated, which bears the potential to reduce suboptimal responses and reduce toxicity related to high exposure [17]. Moreover, intratumoral overexpression of CYP3A has been reported as a possible detoxification or resistance mechanism in many different types of cancer, including breast and ovarian cancer compared to healthy tissue [18,19]. Theoretically, inhibition of CYP3A by the “booster agent” may lead to higher intratumoral concentrations of the drug and may subsequently result in a more effective PARP-inhibition in tumour cells [20].

Recently, the proof-of-concept PROACTIVE-A study has shown that PK boosting of olaparib is feasible. The boosted therapy of olaparib 100 mg twice daily (BID) with the potent CYP3A-inhibitor cobicistat 150 mg BID increased olaparib exposure 1.45-fold compared to the standard therapy of olaparib 300 mg BID [21]. In PROACTIVE-A, patients received the boosted therapy for one week, precluding conclusions on efficacy and longer-term tolerability.

The ongoing PROACTIVE-B study aims to investigate the efficacy and tolerability of the boosted olaparib treatment compared to the standard monotherapy with the aim to implement the boosting approach for olaparib in routine clinical practice.

## 2 Methods

### 2.1 Design

**Fig. 1:**
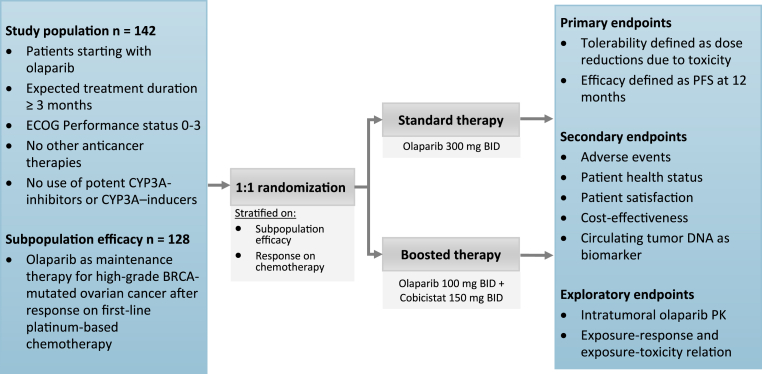
Study design of the PROACTIVE-B study. BRCA: Breast Cancer gene; CYP3A: Cytochrome P450 3A enzymes; ECOG: Eastern Cooperative Oncology Group; PK: Pharmacokinetics; PFS: Progression-free survival.

PROACTIVE-B is a Dutch nationwide, open-label, randomised, multicentre trial comparing boosted olaparib therapy with standard dose olaparib monotherapy in patients with solid tumours (Fig. 1). Patients will be randomised 1:1 between standard olaparib monotherapy and the boosted therapy of olaparib and cobicistat by a variable block randomisation. Randomisation will be stratified by tumour type (BRCA-mutated, high-grade, serous ovarian cancer treated with olaparib after first-line platinum-based chemotherapy, or other) and response to chemotherapy (i.e., complete, or partial response). The standard full dose of olaparib monotherapy consists of 300 mg BID. The boosted full dose of olaparib consists of 100 mg BID with cobicistat 150 mg BID. Dose reductions will be performed according to Table 1, in line with the drug label and the physician's discretion. Patients will visit the outpatient clinic at baseline, after 4, 8, 12 weeks and thereafter every three months until progression or the end of olaparib therapy, in line with standard clinical care. Patients will continue treatment until they do not longer experience clinical benefit or until the end of treatment according to the drug label and/or treatment guideline. This current protocol is approved by the Medical Research Ethics Committee Oost-Nederland and is conducted in accordance with Good Clinical Practice and the Declaration of Helsinki.

**Table 1:** Olaparib monotherapy and boosted therapy dose levels.

	Standard monotherapy		Boosted therapy		
	Olaparib	Dose density	Olaparib	Cobicistat	Dose density
**Standard dose**	300 mg BID	100 %	100 mg BID	150 mg BID	100 %
**1st dose reduction**	250 mg BID	83 %	150 mg OD	150 mg BID	75 %
**2nd dose reduction**	200 mg BID	67 %	100 mg OD	150 mg BID	50 %

BID: twice daily; OD: once daily.

### 2.2 Objectives

The primary objective of this study is to determine if boosted olaparib therapy is non-inferior to standard monotherapy concerning both tolerability and efficacy. Tolerability will be measured by dose reductions due to toxicity. Efficacy will be measured by progression-free survival (PFS) in patients with high-grade serous BRCA-mutated ovarian cancer treated with olaparib maintenance therapy after first-line platinum-based chemotherapy.

Secondary objectives include establishing potential differences in type of toxicity, patient health status, therapy satisfaction, cost-effectiveness between treatment arms. Moreover, the predictive value of circulating tumour DNA (ctDNA) as a pharmacodynamic response marker will be evaluated. Exploratory objectives include the association between olaparib PK and toxicity and efficacy, and the association between patient characteristics and olaparib PK and efficacy. Finally, differences in intratumoral olaparib concentrations between treatment arms will be explored.

### 2.3 Endpoints and assessments

The co-primary endpoints are dose reductions due to toxicity and PFS, defined as the time from randomisation until the date of either objective radiological disease progression or combined biochemical and clinical progression or death. The reasons for dose reductions will be recorded. Objective radiological disease progression will be assessed according to the Response Evaluation Criteria in Solid Tumours (RECIST) version 1.1, or modified RECIST according to the Gynecologic Cancer Intergroup (GCIG) if there is no measurable disease at baseline [22,23]. Biochemical progression is assessed according to the GCIG criteria for CA-125 and clinical progression will be established by the treating physician [23].

For toxicity, adverse events will be scored according to the Common Terminology Criteria for Adverse Events (CTCAE) version 5.0 [24]. Patient health status and quality of life will be measured with the EuroQol 5 dimensions 5 levels questionnaire (EQ-5D-5L) version 1.1 [25]. Patient satisfaction in terms of cancer therapy will be assessed with the Cancer Therapy Satisfaction Questionnaire (CTSQ) [26]. To measure costs from the societal perspective for the cost-effectiveness analysis, the Institute for Medical Technology Assessment (iMTA) Productivity Cost Questionnaire (iPCQ) and iMTA Medical Consumption Questionnaire (iMCQ) will be used [27,28]. ctDNA levels in plasma will be measured with shallow Whole Genome Sequencing (sWGS) and/or a hybrid-capture based Next Generation Sequencing (NGS) panel, dependent on the most recent insights at time of analysis. ctDNA levels will be corrected for germline and clonal haematopoietic variants with matched peripheral blood mononuclear cells. Olaparib plasma concentrations will be measured by cross-validated liquid chromatography tandem mass-spectrometry assays [21,29,30]. Intratumoral olaparib concentrations will be assessed from optional tumour biopsies. The frequency of the above-mentioned assessments is outlined in Table 2.

**Table 2:** Overview of measurements in PROACTIVE-B study.

	Baseline	Week 4	Week 6	Week 8	Week 12	Every 3 months	Every 6 months	At progression
Clinical evaluation	X	X		X	X	X	X	
Laboratory	X	X		X	X	X	X	
Adverse events	X	X		X	X			
Radiological evaluation	X				X		X	
ctDNA	X	X		X	X			X
Plasma olaparib PK		X		X	X			
Intratumoral olaparib PK				X				X
Online questionnaires	EQ-5D-5L iPCQ iMCQ		CTSQ EQ-5D-5L iPCQ		CTSQ EQ-5D-5L iPCQ iMCQ		CTSQ EQ-5D-5L iPCQ iMCQ	EQ-5D-5L

ctDNA: circulating tumour DNA; CTSQ: Cancer Therapy Satisfaction Questionnaire; EQ-5D-5L: EuroQol 5 dimensions 5 levels questionnaire; iMCQ: iMTA Medical Consumption Questionnaire; iPCQ: iMTA Productivity Cost Question.

### 2.4 Study population

Patients are eligible for this study when the following inclusion criteria are met.

• Age ≥18 year;
• Starting with olaparib treatment, according to the drug label and physician's discretion;
• Expected to be on olaparib treatment for ≥3 months;
• Eastern Cooperative Oncology Group (ECOG) performance status of 0–3;
• Able and willing to provide written informed consent prior to screening;
• Able to measure the outcome of the study in this patient (e.g., patient availability; willing and being able to undergo sample collection).

Patients will be excluded if any of the following exclusion criteria apply.

• Concurrent use of other anticancer therapies;
• Concurrent use of potent inducers or inhibitors of CYP3A4 as assessed with the Dutch database *KNMP G-standard;*
• Known contra-indications for cobicistat according to the Summary of Product Characteristics (e.g., concomitant use of alfuzosin, carbamazepine, phenytoin, colchicine, rifampin).

In total, 128 of the 142 patients should receive olaparib as maintenance therapy for BRCA-mutated, FIGO III/IV ovarian cancer after response on first-line platinum-containing chemotherapy for the evaluation of efficacy in a homogenous population. The remaining 14 patients should receive olaparib for any approved indication of olaparib according to the drug label, to illustrate the extrapolation of the boosting concept in other populations. These 14 patients are pooled with the 128 patients with ovarian cancer for the evaluation of tolerability as the incidence of dose reductions due to adverse reactions is theoretically similar across tumour types [4,[31], [32], [33], [34]].

Patients may decide to withdraw from the study at any time. Patients without an event, who withdraw within three months after start will be replaced by a new eligible patient. Replaced patients will be monitored to prevent bias, a maximum of ∼30 % can be replaced.

### 2.5 Participating sites

In the Netherlands, patients with ovarian cancer are discussed in a regional multidisciplinary tumour board, with a participating site of the PROACTIVE-B, before initiating treatment. The study is executed in 10 hospitals in the Netherlands: Amphia Hospital, Breda; Amsterdam University Medical Centres, Amsterdam; Antoni van Leeuwenhoek – Netherlands Cancer Institute, Amsterdam; Erasmus University Medical Centre, Rotterdam; Jeroen Bosch Hospital, ‘s Hertogenbosch; Leiden University Medical Centre, Leiden; Maastricht University Medical Centre+, Maastricht; Radboud University Medical Centre, Nijmegen; University Medical Centre Groningen, Groningen; University Medical Centre Utrecht, Utrecht. Currently, all sites are open for inclusion.

### 2.6 Sample size calculation

Separate sample size calculations were performed for the two primary endpoints tolerability and efficacy. The type I error of 0.05 was distributed between both outcomes.

For non-inferiority of tolerability, the non-inferiority margin for dose reductions due to toxicity was set at 24 %. With a type I error of 0.01, power of 80 %, and an incidence of dose reductions of 28 % in each group [31], 71 patients need to be randomised to each treatment group. The non-inferiority margin for tolerability of 24 % is relatively large. This has been extensively discussed with the clinicians and patient advocates experienced in the treatment with olaparib. The most prominent adverse events related to olaparib therapy are anaemia and gastro-intestinal symptoms [35]. Since patients who start olaparib are initially closely monitored, these adverse events are usually detected shortly after start of olaparib and are easily and effectively managed with dose reductions. Therefore, the clinical impact of these side effects is acceptable, and the larger non-inferiority margin of 24 % was accepted.

For non-inferiority of efficacy, the non-inferiority margin for PFS rate at twelve months was set at 15 %. With a type I error of 0.04, power of 80 %, and an estimated PFS at twelve months of 88 % in each group, 64 patients need to be randomised to each treatment group [31]. The non-inferiority margin for efficacy of 15 % was set according to the EMA guideline [36]. The margin demonstrates superiority to placebo as olaparib increased progression-free survival at 12 months by 33 % compared to placebo [31]. Moreover, the non-inferiority margin has been discussed and endorsed by clinicians and patient advocates.

Thus, the total sample size was set at 142 patients, of which 128 will receive olaparib for high-grade, BRCA-mutated ovarian cancer after response on first-line platinum-based chemotherapy.

### 2.7 Statistical analysis

An intention-to-treat, and per protocol, analysis will be performed in line with guidelines. The per-protocol analysis will be leading, but it is not expected that cross-over will take place. Proportions of patients with dose reductions due to toxicity at twelve months will be compared between both treatment arms with the Chi-square test for proportions. Additionally, a Kaplan-Meier approach will be used to compare PFS rates between both treatment arms in the efficacy population.

Linear mixed-effects models will be used to evaluate differences in patient health status and therapy satisfaction over time and between treatment arms. Descriptive statistics will be used to describe adverse events and intratumoral olaparib concentration. The association between ctDNA levels and clinical outcomes (PFS/OS) will be explored with a Cox proportional hazard model with a correction for previously defined prognostic factors [37]. Additionally, cost-effectiveness will be analysed in line with the most recent guideline for economical evaluations in healthcare by the Dutch Healthcare Institute [38]. In line with this guideline, we will adopt a societal perspective and a life time horizon to calculate the costs per quality adjusted life year (QALY) in a cost-utility analysis [38].

The patterns and extend of missing data over time and between treatment arms will be explored. As the primary endpoints are easy to assess, missing data is expected to be minimal for the primary analysis. In case of unexpected high proportions of missing data or missing data that can be classified as Missing Not At Random (MNAR), imputation methods and/or sensitivity analyses will be applied [39].

## 3 Discussion

The PROACTIVE-B study aims to demonstrate non-inferiority of olaparib treatment by boosting a reduced dose with cobicistat. This could result in reduced variability in exposure and toxicity. Moreover, it will reduce the expenses related to olaparib therapy without compromising efficacy.

At the base of the study is the proof-of-concept PROACTIVE-A study. In this cross-over study, boosted olaparib therapy resulted in higher olaparib plasma exposure than the standard monotherapy [21]. Based on the principle that drug exposure is related to effect, it is expected that the boosted therapy will lead to at least similar efficacy. However, as PK boosting is a new concept in the field of oncology, it was concluded that it is desirable to establish similar efficacy of the boosted strategy in at least one patient population, for broad acceptance and implementation of the concept.

It is uncertain if the boosted therapy will lead to increased adverse events due to higher olaparib plasma exposure, which was seen in PROACTIVE-A. Olaparib toxicity is related to exposure, and therefore higher exposure levels might lead to increased adverse events [17]. However, in the PROACTIVE-A, patients who tolerated olaparib well were included. This population had approximately 67 % lower olaparib exposure at the standard dose compared to previous studies [21]. The low olaparib exposure is attributed to higher CYP3A-activity and thus, higher clearance of olaparib [21]. We hypothesized that olaparib exposure will be normalized with the boosted therapy, due to reduced PK variability. Therefore, patients with higher CYP3A-acitivity and low olaparib exposure would have a more pronounced effect of boosting, corresponding with a greater increase in olaparib exposure. Simultaneously, the effect of boosting would be less pronounced in patients with an average or high exposure at the standard dose of olaparib. These patients, who are already at risk for toxicity with a high exposure, might experience a less pronounced effect of PK boosting. In the current trial, patients will be included before start of olaparib, and the risk of selection bias is reduced. Therefore, this trial will provide valuable insights into the effect of PK boosting on olaparib exposure levels and adverse events across a representative patient population.

To ensure feasibility of the protocol, this study is aligned with the current standard clinical practice as much as possible. Additional follow-up visits outside the study protocol are accepted and monitored for adverse event reporting as well. Questionnaires will be sent out online to reduce burden for the clinic and research staff, but also to make it as convenient as possible for patients who can fill in the questionnaires at a moment that fits them best.

The wide inclusion criteria of the study will result in a study population that reflects the real-world population. This will lead to broad external validity of our results. Of note, there is no exclusion criterion for patients treated with comedication that are substrates for CYP3A. These drugs could also be affected by cobicistat and, therefore, patients are screened for drug-drug interactions through routine workflows. Relevant interactions may limit the use of cobicistat. Due to the extensive experience with these drugs in antiretroviral therapy, this is considered a manageable hurdle [40].

Besides the clinical evaluation of the boosted therapy, this study incorporates several emerging research topics. In the past years, ctDNA analysis have provided valuable insights in its clinical validity in several cancer types [41]. In patients with ovarian cancer, ctDNA is correlated with the volume of disease and holds promise as a biomarker for disease monitoring [42,43]. This is interesting, considering the poor concordance between CA-125 and radiological progression in patients treated with PARP-inhibitors [44]. Moreover, reversion mutations could be detected in ctDNA of patients after treatment with olaparib and cediranib, which could provide valuable insights for treatment optimization [45].

Also, tumour biopsies will shed light on the intratumoral olaparib concentration, which might be increased with the boosted therapy and could therefore be a potential tool to convert resistance. Preclinical studies have shown that tumours are able to express CYP3A as a detoxification mechanism [18,20]. By inhibiting CYP3A, PK boosting might even increase intratumoral olaparib concentrations by CYP3A-inhibition and could therefore improve efficacy. The tumour biopsies are optional in this study, but even a small number of biopsies will provide new insights.

The PROACTIVE-B study is a post-approval fully academic clinical trial that aims to improve the cost-effectiveness of olaparib. In an era of rising costs of anticancer drugs, it is of utmost importance that these investigator-initiated studies are performed as they can significantly improve treatment for patients, while simultaneously reduce healthcare expenditures.

## CRediT authorship contribution statement

**Joanneke K. Overbeek:** Writing – original draft, Investigation, Formal analysis. **Niels A.D. Guchelaar:** Writing – review & editing, Investigation. **Ma Ida Mohmaed Ali:** Writing – review & editing, Investigation. **Muriëlle Sark:** Writing – review & editing, Methodology. **Carolien Hovenier:** Writing – review & editing, Methodology. **Wietske Kievit:** Writing – review & editing, Methodology, Formal analysis, Conceptualization. **Marjolijn J.L. Ligtenberg:** Writing – review & editing, Methodology, Formal analysis, Conceptualization. **Petronella B. Ottevanger:** Writing – review & editing, Methodology, Investigation, Conceptualization. **Haiko J. Bloemendal:** Writing – review & editing, Methodology, Investigation, Conceptualization. **Stijn L.W. Koolen:** Writing – review & editing, Methodology, Conceptualization. **Ron H.J. Mathijssen:** Writing – review & editing, Methodology, Investigation, Conceptualization. **Ingrid A. Boere:** Writing – review & editing, Methodology, Investigation, Conceptualization. **Alwin D.R. Huitema:** Writing – review & editing, Methodology, Conceptualization. **Gabe S. Sonke:** Writing – review & editing, Methodology, Investigation, Conceptualization. **Frans L. Opdam:** Writing – review & editing, Methodology, Investigation, Conceptualization. **Rob ter Heine:** Writing – review & editing, Methodology, Funding acquisition, Formal analysis, Conceptualization. **Nielka P. van Erp:** Writing – review & editing, Methodology, Investigation, Funding acquisition, Conceptualization.

## Ethics approval and consent to participate

The study protocol has been reviewed and approved by the Medical Research Ethics Committee Oost-Nederland (reference number NL78695.091.21). In addition, the study has been reviewed and approved by local ethics committees of participating centres. The study will be conducted in accordance with the International Conference on Harmonisation: Good Clinical Practice and the Declaration of Helsinki. Patients will be informed and asked for written consent by their treating physician, local investigator, or research nurse. Separate written informed consent will be asked for the optional tumour biopsies.

## Funding

This work is funded by 10.13039/501100001826ZonMw, The 10.13039/501100001826Netherlands Organisation for Health Research and Development, as part of the *Goed Gebruik Geneesmiddelen* program (grant number 10140021910005). Additional funding is provided by 10.13039/501100012524Netherlands Federation of University Medical Centres as part of the *Transformatiedeal*. These subsiding parties had no influence on the design of the study or writing of the manuscript.

## Declaration of competing interest

The authors declare the following financial interests/personal relationships which may be considered as potential competing interests:

MJLL reports consulting fees paid to the institution from 10.13039/100004325AstraZeneca, 10.13039/100004330GlaxoSmithKline, and 10.13039/100015756Janssen-Cilag and speakers fees paid tot the institution from Roijé congressen and Uitgeverij Jaap. RHJM reports research funding paid to the institute from Astellas, 10.13039/100004326Bayer, Boehringer-Ingelheim, Cristal Therapeutics, Deuter Oncology, Echo Pharmaceuticals, 10.13039/501100014812Nordic Pharma, 10.13039/100004336Novartis, Pamgene, 10.13039/100004319Pfizer, 10.13039/100004337Roche, 10.13039/100004339Sanofi, and 10.13039/501100011725Servier. IAB: reports research funding paid to the institute from 10.13039/100004330GlaxoSmithKline. GSS: reports research funding paid to the institute from 10.13039/100022874Agendia, 10.13039/100004325AstraZeneca, 10.13039/100004334Merck, 10.13039/100004336Novartis, 10.13039/100004337Roche, and 10.13039/100020124Seagen and a consulting role for Biovica and 10.13039/100020124Seagen. FLO: reports research funding from 10.13039/100002429Amgen, 10.13039/100004325AstraZeneca, 10.13039/100030732MSD, 10.13039/100020124Seagen, 10.13039/100004337Roche, Cytovation, Revolution Medicine, Ascendis Pharma and consulting fees from Iomx. RtH: reports research funding from 10.13039/100002429Amgen and consulting fees from Academic Medical Education and 10.13039/100004358Samsung Bioepis. NPvE: reports research funding paid to the institute from 10.13039/100004324Astellas and 10.13039/501100014382Ipsen. JKO, NADG, MIMA, MS, CH, WK, PBO, HJB, SLWK, ADRH declare no potential conflicts of interest.

## Data Availability

No data was used for the research described in the article.
